# Pareto-Front Optimization of Variance-Added Expected Loss with Interrelated Qualities

**DOI:** 10.3390/e28020199

**Published:** 2026-02-10

**Authors:** Sangwon Kim, Kichun Lee

**Affiliations:** Department of Industrial Engineering, Hanyang University, Seoul 04763, Republic of Korea; genis0627@naver.com

**Keywords:** multivariate loss function, Pareto frontier, variance loss, trade-off analysis

## Abstract

In industries, particularly in quality optimization, the trade-off between model bias and variance is inevitable, reflecting the tension between accuracy and uncertainty. Traditional methods often address these aspects separately, potentially leading to suboptimal decisions. This study proposes a Pareto-front optimization framework for a variance-added expected loss function within the context of interrelated quality characteristics. By integrating multivariate quadratic loss with a variance term, our approach simultaneously captures deviation from targets (bias) and system uncertainty (variance). Unlike sequential approaches that first minimize bias and then variance—often increasing total risk—our weighted formulation flexibly adjusts for their trade-offs. This enables a more balanced and efficient optimization process that identifies solutions with lower overall risk. Through Pareto-front analysis, we reveal trade-offs between expected loss and variance, allowing users to select optimal quality designs based on their preferred bias–variance balance. Representative examples and a case study adopted from the literature validate the effectiveness and practical applicability of the proposed method.

## 1. Introduction

In modern industries, achieving optimal performance across multiple interrelated quality attributes is a complex, yet essential, task. Systems often face conflicting objectives, in which improving one quality indicator may deteriorate another. As industries demand higher precision and robustness, efficiently managing these trade-offs becomes increasingly critical. In a machine-learning task, for example, a trade-off between model bias and model variance is inevitable: a low-level model bias from substantial model complexity incurs high-level model variance, and vice versa. Representative quality objectives in conflict are accuracy and variance arising from predefined targets and random observations.

Traditional optimization methods typically focus on minimizing a single objective loss function, but they often fail to account for interdependencies between different quality attributes. Many real-world systems such as manufacturing, engineering, and healthcare exhibit tightly coupled quality characteristics that directly influence overall system performance. In such contexts, a more comprehensive approach is required—one that considers not only deviations from target quality but also uncertainty in system behavior by incorporating variance into overall loss.

This paper proposes a novel approach for globally optimizing interrelated quality attributes by adding variance-induced uncertainty to expected loss. The goal is to minimize not only deviation from a target quality but also uncertainty in performance. For the sake of global optimization, we use Pareto fronts to explore trade-offs between conflicting objectives, enabling decision-makers to select solutions, or decisions, that achieve the desired balance between accuracy and risk. Importantly, our method allows users to explicitly choose the ratio between bias and variance terms, providing greater flexibility to manage the trade-offs arising from specific application requirements.

In this study, we apply an alternative global criterion (GC)-based method for generating Pareto fronts to handle accuracy-induced and variance-induced qualities. This method offers increased computational efficiency compared to traditional optimization techniques and effectively handles the trade-offs between multiple objectives. We illustrate the applicability of this approach through two representative examples and demonstrate its practical use with a dataset adopted from the literature. The experiments reveal the applicability of the proposed method to a complex system with interrelated quality attributes. Our analysis shows that the optimization results outperform existing quality decisions in terms of both quality and flexibility, providing valuable insights into achieving quality optimization in industrial settings.

The proposed method presented in this paper offers a systematic framework for quality optimization in complex systems with interrelated attributes and variance. By applying this approach, decision-making processes in industries requiring simultaneous optimization of multiple quality factors can be significantly improved.

## 2. Related Works

In multi-objective optimization problems, it is often necessary to simultaneously consider distinct quality characteristics such as mean deviation and variance. As a result, conventional single-objective optimization techniques have limited applicability, and various multi-objective quality optimization methods have been proposed.

A well-known early approach is the two-step strategy proposed by [1], in which variance is minimized first, followed by minimization of mean deviation. This method is computationally simple and easy to interpret, making it appealing in practical settings. However, in real-world applications, most design variables influence both the mean and variance simultaneously. Thus, separating these objectives can lead to inaccurate decisions and reduce the overall predictive accuracy and interpretability of the optimization process.

Other studies have sought to mathematically incorporate interactions between multiple quality characteristics. For example, Shiau proposed a weighted sum of signal-to-noise (SN) ratios to aggregate multiple quality characteristics [2], while Tong et al. developed a multi-response SN ratio that sums weighted expected losses before converting the result to an overall performance measure [3]. More recently, a loss function explicitly considering interactions between variables has been proposed (see, e.g., [4]), offering a more theoretically robust formulation compared to earlier methods. However, these approaches share practical limitations: they rely mainly on numerical computations from existing data, do not support the setting of design variables or the generation of actionable design alternatives, and thus are generally restricted to post hoc analysis rather than active design optimization.

Building on such interaction-based loss functions, Costa and Lourenço [5] proposed a game-theoretic approach to simultaneously optimize process and product quality characteristics in bi-objective settings. By employing Stackelberg’s strategy, their method avoids subjective parameter tuning and seeks efficient trade-offs. Nonetheless, such game-theoretic frameworks often struggle to yield a solution that simultaneously satisfies all quality objectives, especially when multiple interdependent characteristics are involved. This underscores the need for methods that not only capture interactions among characteristics but also offer clear and actionable design recommendations.

To address such multi-objective optimization challenges, especially in design settings, surrogate-based approaches have gained popularity. A representative example is the efficient global optimization (EGO) algorithm [6], which uses the expected improvement (EI) criterion to sequentially explore the design space and identify optima. Ref. [7] extended this framework to multi-objective problems by introducing ParEGO, an algorithm that employs Tchebycheff scalarization to search the Pareto front. Furthermore, ref. [8] proposed an integrated approach combining multiple scalarization techniques (e.g., Tchebycheff and weighted sum) to better approximate diverse trade-offs. However, these methods inherently reduce multi-objective problems to a series of single-objective subproblems and, as a result, cannot fully capture the complex trade-offs among conflicting objectives.

In this study, we propose a multivariate quadratic loss-based Pareto optimization framework that mathematically captures the interactions between design variables and multiple quality characteristics, and simultaneously accounts for overall quality loss arising from both mean deviation and variance. In contrast to scalarization-based methods, the proposed approach directly derives interpretable Pareto-optimal solutions in the multi-objective space without reducing the problem to a single-objective formulation. For reproducibility, we provide a GitHub URL (https://github.com/KIM-sang-won/PFOVELIQ/blob/main/PFOVE__Python_code.ipynb, accessed on 4 February 2026) for the experiments.

## 3. Methods

### 3.1. Multivariate Quadratic Loss Function and Total Risk

A multivariate quadratic loss function was introduced by Pignatiello [1] and is defined as follows:

(1) $$L\left(y\right)={\left(y-\tau \right)}^{T}C\left(y-\tau \right),$$

where $y={\left(y_{1},\ldots ,y_{p}\right)}^{T}$ is a p×1 vector consisting of principal components (PCs) of the ’nominal-the-best’ type, and $\tau ={\left(\tau_{1},\ldots ,\tau_{p}\right)}^{T}$ is a p×1 vector containing the corresponding target values. The p×p positive definite cost matrix C assigns weights to deviations among different PCs.

The elements of C are determined by defining the loss in terms of deviations. Let $z_{i}=y_{i}-\tau_{i}$ denote the deviation of the *i*-th characteristic from its target. For example, in a bivariate case (p=2), the loss function is expressed as:

$$L\left(y\right)=c_{11}z_{1}^{2}+c_{22}z_{2}^{2}+2c_{12}z_{1}z_{2},$$

in which $c_{ii}$ represents the cost coefficient associated with the squared deviation $z_{i}^{2}$, and $c_{ij}$, i≠j, accounts for the interaction effect between deviations $z_{i}$ and $z_{j}$. By default, y is of the nominal-the-best type, but it can be adjusted to the lower-the-better or higher-the-better type by modifying τ accordingly.

Furthermore, Pignatiello [1] assumes that y follows a *p*-dimensional multivariate normal distribution with mean vector $\mu ={\left(\mu_{1},\ldots ,\mu_{p}\right)}^{T}$ and p×p variance–covariance matrix $\Sigma =(\sigma_{ij}^{2})$, i.e.,

(2) y∼N(μ,Σ),

where μ represents the expected values of the PCs, and Σ captures their variability and correlations.

The expected quadratic loss can be decomposed into bias and variance contributions:

(3) $$E\left[L\left(y\right)\right]={\left(\mu -\tau \right)}^{T}C\left(\mu -\tau \right)+\mathrm{trace}\left(C\Sigma \right),$$

where the first term measures the deviation of the mean from the target, and the second term represents the variance contribution weighted by C.

We define the total risk *T* by adding the variance contribution weighted by the parameter λ:

(4) $$T=E\left[L\left(y\right)\right]+\lambda \;\mathrm{trace}\left(C\Sigma \right)={\left(\mu -\tau \right)}^{T}C\left(\mu -\tau \right)+\left(1+\lambda \right)\;\mathrm{trace}\left(C\Sigma \right),$$

where we used $E\left[L\left(y\right)\right]={\left(\mu -\tau \right)}^{T}C\left(\mu -\tau \right)+\mathrm{trace}\left(C\Sigma \right)$. The term trace(CΣ) derives from the identity

$$E\left[{\left(y-\mu \right)}^{T}C\left(y-\mu \right)\right]=\mathrm{trace}\left(E\left[{\left(y-\mu \right)}^{T}C\left(y-\mu \right)\right]\right)=\mathrm{trace}\left(E\left[C\left(y-\mu \right){\left(y-\mu \right)}^{T}\right]\right)=\mathrm{trace}\left(C\Sigma \right).$$

Physically, it represents cost-weighted variability, implying that variance contributes to loss only when associated with non-zero costs in C. Thus, the risk is interpreted in terms of economic consequences rather than variance alone. The parameter λ adjusts this variance contribution, typically satisfying λ≥0 but allowing λ≥−1.

Mathematically, introducing λ generalizes the standard expected loss by relaxing the rigid 1:1 bias–variance trade-off, allowing for a flexible control and scalarization of conflicting objectives. Practically, minimizing variance is often more critical than centering the mean, as excessive variability can lead to irreversible consequences. For instance, high variance can cause matching failures in precision assembly or explosion risks in chemical processes, whereas mean shifts are often correctable. Thus, λ allows for heavier penalization of variability, reflecting a risk-averse strategy in robust design.

### 3.2. Risk Derivation for Mixed Quality Characteristics

To accommodate both nominal-the-best ($\tau_{i}\neq 0$) and smaller-the-better ($\tau_{i}=0$) types of quality characteristics, we partition the vector y into two sub-vectors, $y_{1}$ and $y_{2}$:

$$y={\left[y_{1}^{T}\;\;y_{2}^{T}\right]}^{T},$$

where $y_{1}={\left(y_{1},\ldots ,y_{q}\right)}^{T}$ is a q×1 vector of nominal-the-best PCs, and $y_{2}={\left(y_{q+1},\ldots ,y_{p}\right)}^{T}$ is a (p−q)×1 vector of smaller-the-better PCs. For simplicity, one may also scale y to remove unit effects in the loss function.

Following this partition, all related vectors and matrices are restructured accordingly. In particular, the mean vector μ and the target vector τ are expressed as

$$\mu ={\left[\mu_{1}^{T}\;\;\mu_{2}^{T}\right]}^{T},\;\tau ={\left[\tau_{1}^{T}\;\;\tau_{2}^{T}\right]}^{T},\;\mathrm{with}\;\tau_{2}=0.$$

The covariance matrix Σ and the cost matrix C are also expressed in block forms, respectively:

$$\Sigma =\left[\begin{array}{cc} \Sigma_{11} & \Sigma_{12}\\ \Sigma_{12}^{T} & \Sigma_{22} \end{array}\right],\;C=\left[\begin{array}{cc} C_{11} & C_{12}\\ C_{12}^{T} & C_{22} \end{array}\right].$$

Using this structure, the multivariate quadratic loss function for y is defined as

$$\begin{array}{cc} L(y) & ={\left(y-\tau \right)}^{T}C\left(y-\tau \right)={\left[\begin{array}{c} y_{1}-\tau_{1}\\ y_{2} \end{array}\right]}^{T}\left[\begin{array}{cc} C_{11} & C_{12}\\ C_{12}^{T} & C_{22} \end{array}\right]\left[\begin{array}{c} y_{1}-\tau_{1}\\ y_{2} \end{array}\right]\\ & ={\left(y_{1}-\tau_{1}\right)}^{T}C_{11}\left(y_{1}-\tau_{1}\right)+y_{2}^{T}C_{22}y_{2}+2{\left(y_{1}-\tau_{1}\right)}^{T}C_{12}y_{2}. \end{array}$$

The expected multivariate quadratic loss of y can then be written as

$$E\left[L\left(y\right)\right]=E\left[{\left(y_{1}-\tau_{1}\right)}^{T}C_{11}\left(y_{1}-\tau_{1}\right)\right]+E\left[y_{2}^{T}C_{22}y_{2}\right]+2\;E\left[{\left(y_{1}-\tau_{1}\right)}^{T}C_{12}y_{2}\right].$$

Expanding the first and second terms using the mean vector μ and variance–covariance matrix Σ, we have

$$\begin{array}{cc} E\left[{\left(y_{1}-\tau_{1}\right)}^{T}C_{11}\left(y_{1}-\tau_{1}\right)\right] & =\mathrm{trace}\left(C_{11}\Sigma_{11}\right)+\mathrm{trace}(C_{11}\left(\mu_{1}-\tau_{1}\right){\left(\mu_{1}-\tau_{1}\right)}^{T}),\\ E\left[y_{2}^{T}C_{22}y_{2}\right] & =\mathrm{trace}\left(C_{22}\Sigma_{22}\right)+\mathrm{trace}\left(C_{22}\mu_{2}\mu_{2}^{T}\right), \end{array}$$

and the third term can be rewritten as

$$E\left[{\left(y_{1}-\tau_{1}\right)}^{T}C_{12}y_{2}\right]=\mathrm{trace}\left(C_{12}\Sigma_{12}^{T}\right)+\mathrm{trace}\left(\mu_{2}{\left(\mu_{1}-\tau_{1}\right)}^{T}C_{12}\right).$$

Combining all three terms, the expected loss becomes

(5) $$\begin{array}{cc} E[L(y)] & =\mathrm{trace}\left(C_{11}\Sigma_{11}\right)+\mathrm{trace}(C_{11}\left(\mu_{1}-\tau_{1}\right){\left(\mu_{1}-\tau_{1}\right)}^{T})\\ & \;+\mathrm{trace}\left(C_{22}\Sigma_{22}\right)+\mathrm{trace}\left(C_{22}\mu_{2}\mu_{2}^{T}\right)\\ & \;+2\;\mathrm{trace}\left(C_{12}\Sigma_{12}^{T}\right)+2\;\mathrm{trace}\left(\mu_{2}{\left(\mu_{1}-\tau_{1}\right)}^{T}C_{12}\right)\\ & =\mathrm{trace}\left(C\left(\mu -\tau \right){\left(\mu -\tau \right)}^{T}\right)+\mathrm{trace}\left(C\Sigma \right). \end{array}$$

This can also be expressed in summation form as

(6) $$E\left[L\left(y\right)\right]=\sum_{i=1}^{p}\sum_{j=1}^{p}c_{ij}\left(\mu_{i}-\tau_{i}\right)\left(\mu_{j}-\tau_{j}\right)+\sum_{i=1}^{p}\sum_{j=1}^{p}c_{ij}\sigma_{ij}^{2}.$$

Hence, from Equations (5) and (6), we could express the total risk, *T*, as 

(7) $$\begin{array}{cc} T & =E\left[L\left(y\right)\right]+\lambda \mathrm{trace}\left(C\Sigma \right)=\mathrm{trace}\left(C\left(\mu -\tau \right){\left(\mu -\tau \right)}^{T}\right)+\left(1+\lambda \right)\;\mathrm{trace}\left(C\Sigma \right)\\ & =\sum_{i=1}^{p}\sum_{j=1}^{p}c_{ij}\left(\mu_{i}-\tau_{i}\right)\left(\mu_{j}-\tau_{j}\right)+\left(1+\lambda \right)\sum_{i=1}^{p}\sum_{j=1}^{p}c_{ij}\sigma_{ij}^{2}. \end{array}$$

Assuming that independent input variables $x={\left(x_{1},\ldots x_{k}\right)}^{T}$ influence y, the mean vector and covariance matrix are expressed as functions of x, μ(x) and Σ(x). The bias and variance components are then defined as

(8) $$\begin{array}{cc} f_{1}\left(x\right) & =\mathrm{trace}\left(C\left(\mu \left(x\right)-\tau \right){\left(\mu \left(x\right)-\tau \right)}^{T}\right), \end{array}$$

(9) $$\begin{array}{cc} f_{2}\left(x\right) & =\mathrm{trace}\left(C\Sigma (x)\right), \end{array}$$

so that the total risk can be written as $T=f_{1}\left(x\right)+\left(1+\lambda \right)f_{2}\left(x\right)$. Naturally, the total risk T≥0 leads to the parameter, the variance weight, λ≥−1.

The determination of the weighting parameter, 1+λ, is possible in two ways depending on the decision-maker’s strategy. Subjectively, users may assign a value to 1+λ reflecting their perceived importance of bias versus variance. Alternatively, for an objective criterion, 1+λ can be derived from economic cost structures. If we define the total expected cost as $E\left[\mathrm{Cos}t\right]=C_{\mathrm{bias}}f_{1}\left(x\right)+C_{\mathrm{var}}f_{2}\left(x\right)$, where $C_{\mathrm{bias}}$ and $C_{\mathrm{var}}$ are the aggregate cost coefficients for bias and variance respectively, then normalizing the equation by $C_{\mathrm{bias}}$ yields $1+\lambda =C_{\mathrm{var}}/C_{\mathrm{bias}}$. For example, $C_{\mathrm{bias}}$ may represent process adjustment costs, while $C_{\mathrm{var}}$ corresponds to the scrap costs associated with defective products. This formulation allows the trade-off to be driven by tangible economic data when available.

### 3.3. Multi-Objective Optimization via Global Criterion

Ideally, one would minimize the sum of the functions for a given λ. Practically, however, minimizing both functions simultaneously is challenging. For example, field engineers typically determine a few inputs in x to optimize $f_{1}$, then determine the remaining to optimize $f_{2}$ sequentially.

To effectively balance both terms of bias and variance and to globally optimize them, we utilize a Pareto front that represents the comprehensive optimal trade-offs. Unlike standard optimization problems targeting a single objective, multi-objective optimization seeks a set of solutions, called a Pareto optimal set, where no solution is strictly better than the others in all objectives. These solutions form a Pareto front when visualized, allowing us to analyze and adjust λ for an appropriate compromise.

In essence, the Global Criterion (GC) method transforms the multi-objective problem into a scalar minimization problem by measuring the relative deviation of each objective function from its ideal value. We apply an alternative global criterion (GC)-based method for generating a well-distributed Pareto front [9]. The GC function minimizes the distance to a target, defined as $\sum_{i=1}^{n}{\left(|{\hat{y}}_{i}-T_{i}|/(U_{i}-L_{i})\right)}^{p_{i}}$, where ${\hat{y}}_{i}$ is the *i*-th estimated response, $T_{i}$ is the target (here $T_{i}=0$), and $U_{i},L_{i}$ are the bounds.

Adapting this to our problem with two objective functions (n=2), $f_{1}\left(x\right)$ and $f_{2}\left(x\right)$, the GC function is formulated as:

(10) $$\begin{array}{c} \min_{x}\;{\left(\frac{\left|{\hat{f}}_{1}\left(x\right)\right|}{U_{1}-L_{1}}\right)}^{p_{1}}+{\left(\frac{\left|{\hat{f}}_{2}\left(x\right)\right|}{U_{2}-L_{2}}\right)}^{p_{2}}, \end{array}$$

in which the upper ($U_{i}$) and lower ($L_{i}$) bounds are defined as the maximum and minimum values of ${\hat{f}}_{i}\left(x\right)$, an approximation of $f_{i}\left(x\right)$ over the dataset X,

$$U_{i}=\max_{x\in X}{\hat{f}}_{i}\left(x\right),\;L_{i}=\min_{x\in X}{\hat{f}}_{i}\left(x\right).$$

We assume the function is well-behaved enough for its maximum and minimum to be attainable and computable over the dataset X. Alternatively, these bounds can be set by technical constraints or economic conditions. Normalization by $\left(U_{i}-L_{i}\right)$ is crucial for the following reasons [10]:

- It ensures comparability across responses with different scales.
- It reduces subjectivity in weighting.
- It prevents a single criterion from dominating the optimization.
- It improves the spread of Pareto-optimal solutions.

The exponent parameters $p_{1}$ and $p_{2}$ assign priorities to the objectives, enabling the exploration of trade-offs. While $p_{i}$ can theoretically range from 0 to infinity, Costa et al. [9] suggest that $p_{i}>3$ yields diminishing returns. Our experiments confirm that limiting $p_{i}\leq 3$ is sufficient. By varying $p_{1}$ and $p_{2}$ numerically in a grid-search manner, we can explore both convex and non-convex regions of the Pareto frontier [11].

In this research, we perform the optimization over x using pairs of $p_{1}$ and $p_{2}$ from a predefined grid. The resulting diverse set of solutions $x^{*}$ numerically forms the Pareto front, spanning various trade-offs to offer a comprehensive view of the optimization landscape.

To solve Equation (10), we adopt the SLSQP algorithm from Python’s scipy.optimize. minimize library. As a gradient-based method, it efficiently handles equality and inequality constraints, making it well-suited for nonlinear, multi-response problems required to construct an effective Pareto front.

### 3.4. Procedure of Framework

To summarize the operational procedure of our framework, we propose the following four-step workflow. First, appropriate regression models are fitted for the mean, variance, and covariance of the quality characteristics using data from a designed experiment. Second, the bias loss $f_{1}\left(x\right)$ and variance loss $f_{2}\left(x\right)$ are computed as functions of the design variables via the fitted models. Third, the Pareto front is generated by solving the GC minimization problem across a grid of weighting exponents $p_{1}$ and $p_{2}$. Finally, the optimal operating condition is selected from the Pareto front by identifying the point that satisfies a desired risk tolerance or a specific bias-to-variance ratio.

## 4. Experiments

We now present two synthetic cases to demonstrate the applicability of the proposed approach.

### 4.1. Case 1: Bivariate Quality Characteristics (p=2)

In the first case, we consider one NB-type PC and one SB-type PC, i.e., q=1 and p=2:

$$y_{1}=\left(y_{1}\right),\;y_{2}=\left(y_{2}\right).$$

Then, total risk $T=f_{1}\left(x\right)+\left(1+\lambda \right)f_{2}\left(x\right)$ in Equations (7) and (10) is defined by the functions $f_{1}\left(x\right)$ and $f_{2}\left(x\right)$ as follows:

$$f_{1}\left(x\right)=\sum_{i=1}^{2}\sum_{j=1}^{2}c_{ij}\left(\mu_{i}\left(x\right)-\tau_{i}\right)\left(\mu_{j}\left(x\right)-\tau_{j}\right),\;\;\;f_{2}\left(x\right)=\sum_{i=1}^{2}\sum_{j=1}^{2}c_{ij}\sigma_{ij}^{2}\left(x\right).$$

For input variables $x=(x_{1},x_{2})$, $-1\leq x_{i}\leq 1,i=1,2$, let us assume that the estimated regression functions ${\hat{f}}_{1}\left(x\right)$ and ${\hat{f}}_{2}\left(x\right)$, corresponding to $f_{1}\left(x\right)$ and $f_{2}\left(x\right)$, are given as follows:

$${\hat{f}}_{1}\left(x\right)={\left(3x_{1}+3x_{2}+6\right)}^{2},\;{\hat{f}}_{2}\left(x\right)={\left({\left(x_{1}+x_{2}\right)}^{2}+2\right)}^{2}.$$

Furthermore, we set $U_{i}$ and $L_{i}$, for each i=1,2, to be 0 and 100, respectively. The input variables satisfy $-1\leq x_{i}\leq 1$. We increment each parameter of $p_{1}$ and $p_{2}$ from 0.05 to 3 with step size 0.1. The initial starting point in the SLSQP algorithm is set to (0,0).

**Figure 1:**
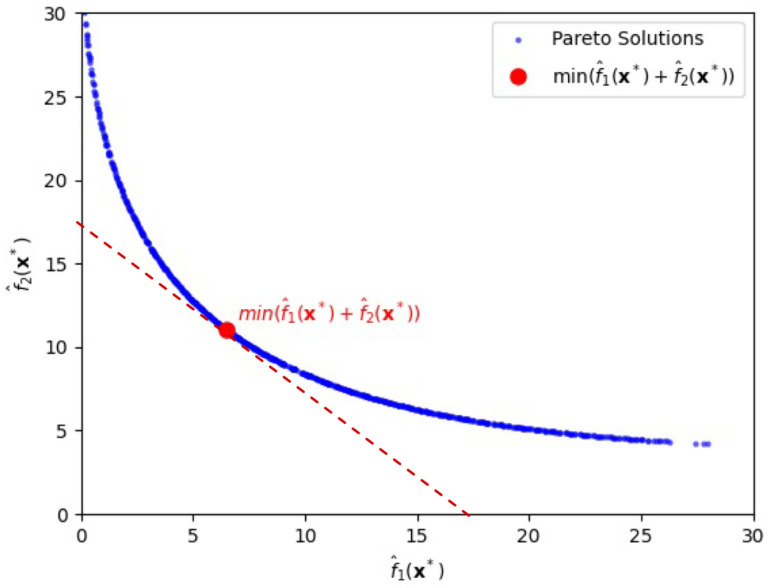
Pareto-front solutions highlighting a solution (depicted by the red circle) that minimizes the sum of bias loss and variance loss, λ=0, for the first case.

Using the alternative GC-based method, we numerically obtain the Pareto frontier shown in Figure 1. In this graph, increasing the value of λ, which reflects a higher emphasis on ${\hat{f}}_{2}\left(x\right)$, leads to solutions x with higher bias but lower variance. Conversely, decreasing λ emphasizes ${\hat{f}}_{1}\left(x\right)$, resulting in lower bias but higher variance.

For instance, as shown in Figure 1, when λ=0, the minimum total risk *T* is 17.54, achieved at $x^{*}=\left(-0.57573,-0.57573\right)$, with corresponding objective values ${\hat{f}}_{1}\left(x^{*}\right)=6.48$ and ${\hat{f}}_{2}\left(x^{*}\right)=11.06$, which is highlighted by the red circle in Figure 1. Notice that for λ=0 the tangent line with the ratio, *x*-intercept/*y*-intercept, being 1 (thereby slope −1) is adopted. If λ differs from zero in general, one can easily draw another tangent line of which the ratio, *x*-intercept/*y*-intercept, is 1+λ (thereby slope −1−λ) in Figure 1, finding corresponding objective values ${\hat{f}}_{1}\left(x^{*}\right)$ and ${\hat{f}}_{2}\left(x^{*}\right)$ along with input vector $x^{*}$.

Here, $x^{*}$ denotes the input vector that minimizes the GC-based objective function for a given value of $p_{i}$, where ${\hat{f}}_{1}\left(x^{*}\right)$ and ${\hat{f}}_{2}\left(x^{*}\right)$ represent the estimated bias and variance components, respectively, at the optimal point. In Figure 1, the red line indicates the tangent with slope −1 to the Pareto frontier at the point where the total risk $T={\hat{f}}_{1}\left(x^{*}\right)+{\hat{f}}_{2}\left(x^{*}\right)$, λ=0, is minimized, illustrating the trade-off between the two objectives. This suggests that decision-makers can adjust the input variables by finding a tangent line with a slope relating to their specific industry characteristics or strategic preferences.

**Figure 2:**
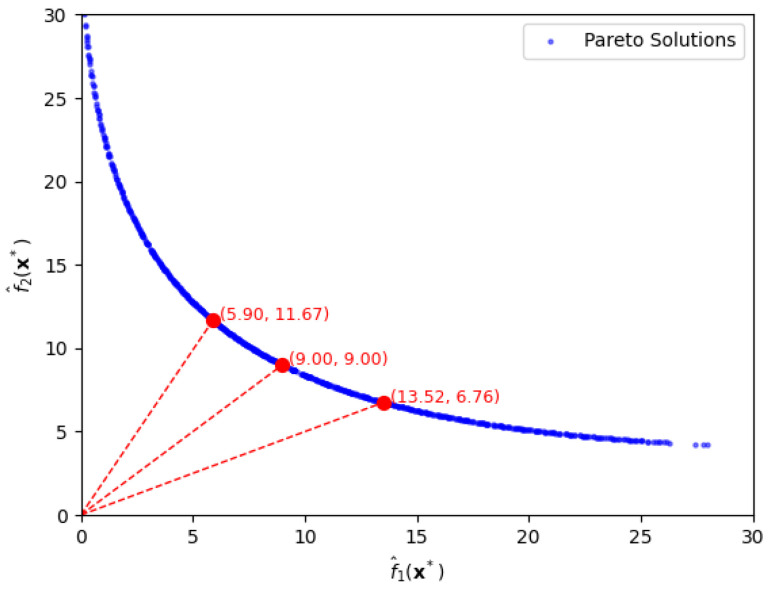
Pareto-front solutions, depicted by the red circles, for specific ratios of bias loss to variance loss, ${\hat{f}}_{1}\left(x^{*}\right):{\hat{f}}_{2}\left(x^{*}\right)$ being 1:2, 1:1, and 2:1, in the first case.

As shown above, the construction of a Pareto front enables flexible control over the trade-off between bias and variance terms. Furthermore, let us suppose that one wants to set a ratio of the bias-loss term to the variance-loss term to a certain value in practice. Figure 2 shows three Pareto-Front solutions for specific ratios, 2:1,1:1, and 1:2 of the two terms under approximation error 0.1.

Firstly, if one sets $f_{1}\left(x\right)$ to be twice $f_{2}\left(x\right)$ with the ratio being 2:1, the optimal solution is x=[−0.387,−0.387], with function values $f_{1}\left(x\right)=13.52$ and $f_{2}\left(x\right)=6.76$. As seen in Figure 2, the solution is easily extracted by drawing a line with slope 1/2 passing through the origin. Secondly, when $f_{1}\left(x\right)$ equals $f_{2}\left(x\right)$, in which the ratio is 1:1, we easily obtain the optimal solution, x=[−0.5,−0.5], which yields function values $f_{1}\left(x\right)=f_{2}\left(x\right)=9.00$. Thirdly, when $f_{1}\left(x\right)$ is half of $f_{2}\left(x\right)$ with a ratio of 1:2, the optimal solution is x=[−0.595,−0.595] with function values $f_{1}\left(x\right)=5.90$ and $f_{2}\left(x\right)=11.67$. These results illustrate how different bias-variance preferences can be effectively represented by specific functional ratios along the Pareto front. We also notice that the extraction of both bias risk $f_{1}\left(x\right)$ and variance risk $f_{2}\left(x\right)$ with a desired ratio is hardly possible without the use of the proposed approach.

### 4.2. Case 2: Four-Variable Quality Characteristics (p=4)

In the second case, we consider the situation where there are two NB-type PCs and two SB-type PCs, i.e., q=2 and p=4:

$$y_{1}=\left(y_{1},y_{2}\right),\;y_{2}=\left(y_{3},y_{4}\right).$$

The total risk $T=f_{1}\left(x\right)+f_{2}\left(x\right)$, λ=0, in Equations (7) and (10) is defined by functions $f_{1}\left(x\right)$ and $f_{2}\left(x\right)$ as follows:

$$f_{1}\left(x\right)=\sum_{i=1}^{4}\sum_{j=1}^{4}c_{ij}\left(\mu_{i}\left(x\right)-\tau_{i}\right)\left(\mu_{j}\left(x\right)-\tau_{j}\right),\;\;\;f_{2}\left(x\right)=\sum_{i=1}^{4}\sum_{j=1}^{4}c_{ij}\sigma_{ij}^{2}\left(x\right).$$

For input variables $x=(x_{1},x_{2},x_{3})$, let us assume that ${\hat{f}}_{1}\left(x\right)$ and ${\hat{f}}_{2}\left(x\right)$ are expressed as follows:

$${\hat{f}}_{1}\left(x\right)={\left(0.5x_{1}^{2}-0.5x_{2}^{2}+0.5x_{3}^{2}+x_{1}x_{3}-4\right)}^{2},\;{\hat{f}}_{2}\left(x\right)={\left(x_{1}^{2}-x_{2}^{2}+3x_{3}\right)}^{2}.$$

**Figure 3:**
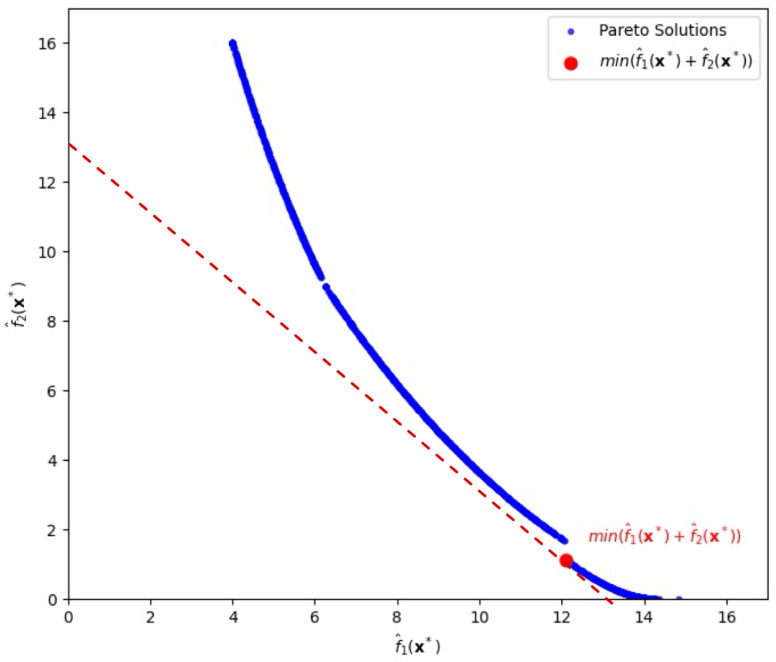
Pareto-front solutions highlighting a solution (depicted by the red circle) that minimizes the sum of bias loss and variance loss, λ=0, for the second case.

Similarly to the first case, the upper bounds of ${\hat{f}}_{1}\left(x\right)$ and ${\hat{f}}_{2}\left(x\right)$ are set to 100, and the lower bounds are set to 0. The initial starting point is (1,1,1), and the input variables are constrained within the range $-1\leq x_{i}\leq 1$. The exponent parameters $p_{i}$ are incremented from 0.05 to 3 with step size 0.05. Under the setting, we obtain a Pareto front as shown in Figure 3.

When λ=0, we achieve the minimum value of the total risk *T* at 13.23314, which is highlighted by the red circle (by slope −1) in Figure 3, with

$${\hat{f}}_{1}\left(x^{*}\right)=12.11403,\;{\hat{f}}_{2}\left(x^{*}\right)=1.11911,$$

and the corresponding input vector is $x^{*}=\left(1,\;0.00209,\;0.01929\right).$

**Figure 4:**
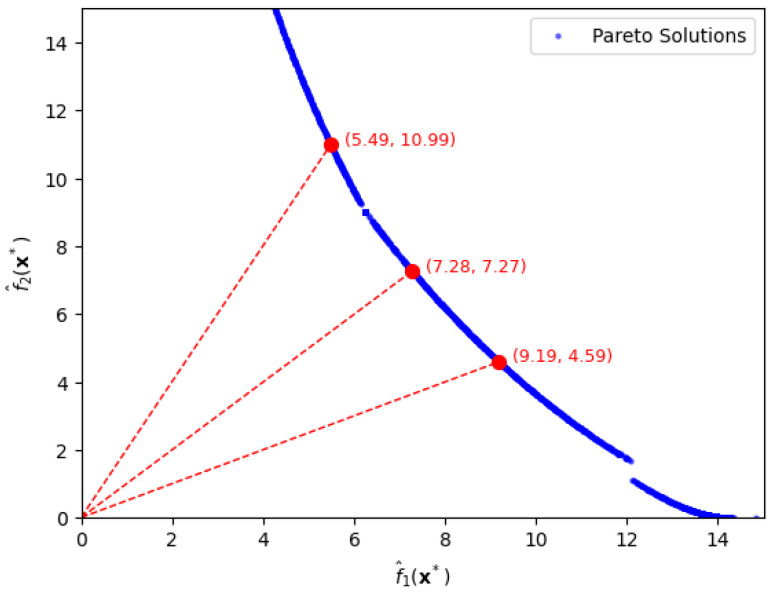
Pareto-front solutions depicted by circles for specific ratios of bias loss to variance loss, ${\hat{f}}_{1}\left(x^{*}\right):{\hat{f}}_{2}\left(x^{*}\right)$ being 1:2, 1:1, and 2:1, in the second case.

By allowing approximation error 0.1, we similarly extract optimal settings under desired trade-off between bias loss and variance loss as shown in Figure 4.

Similarly, we extract the solutions from the Pareto front by drawing a line with the appropriate slope passing through the origin. Firstly, when the ratio $f_{1}\left(x\right)/f_{2}\left(x\right)$ is 2, we find the optimal solution, x=[1.00,1.00,0.71], which yields function values $f_{1}\left(x\right)=9.19,f_{2}\left(x\right)=4.59.$ Secondly, when the ratio $f_{1}\left(x\right)/f_{2}\left(x\right)$ is 1, the optimal solution becomes x=[1.00,1.00,0.90] with function values, $f_{1}\left(x\right)=7.28,f_{2}\left(x\right)=7.27.$ Thirdly, when the ratio $f_{1}\left(x\right)/f_{2}\left(x\right)$ is 1/2, the optimal solution is x=[1.00,0.83,1.00], yielding function values, $f_{1}\left(x\right)=5.49,f_{2}\left(x\right)=10.99.$

We notice that the Pareto frontier obtained using the GC-based method contains visual gaps as shown in Figure 3 and Figure 4. Similar discontinuities have been observed in previous studies [9], which demonstrated that achieving a perfectly connected and comprehensive Pareto front is often difficult, especially in the presence of complex or nonconvex response surfaces.

## 5. Application to Simulation Data

In this section, we apply our approach to a simulation dataset introduced by Pignatiello [1]. This problem serves as a standard test case in multi-response optimization literature, involving two response variables, $y_{1}$ and $y_{2}$, and three input variables, $x_{1},x_{2},$ and $x_{3}$.

### 5.1. Simulation Data and Regression Modeling

The dataset simulates a replicated factorial experiment, enabling the estimation of mean and covariance structures essential for joint optimization. By capturing simultaneous shifts in mean and variance, the Pareto front reveals risk trade-offs that sequential approaches often overlook.

The simulation data is presented in Table 1.

Using the values in each row in Table 1, we obtain the sample mean, sample variance, and sample covariance as shown in Table 2. For example, the first ${\hat{\mu }}_{1}=103.54875$ is the sample average of the four $y_{1}$ observations, 104.454,105.029,99.786,104.923, in Table 1.

Using regression analysis, we derive the following predictive equations:

$$\begin{array}{cc} {\hat{\mu }}_{1}\left(x\right) & =104.9-3.14x_{1}+2.38x_{1}x_{2}-0.35x_{1}x_{3},\\ {\hat{\sigma }}_{11}^{2}\left(x\right) & ={\left(1.613-0.254x_{2}+1.25x_{3}\right)}^{2},\\ {\hat{\mu }}_{2}\left(x\right) & =70.45-0.349x_{1}+3.59x_{2}-0.449x_{1}x_{3}+0.614x_{2}x_{3},\\ {\hat{\sigma }}_{22}^{2}\left(x\right) & ={\left(1.729+0.417x_{1}+1.21x_{3}+0.263x_{1}x_{3}\right)}^{2},\\ {\hat{\sigma }}_{12}^{2}\left(x\right) & =3.712+1.203x_{1}+3.555x_{3}+1.161x_{1}x_{3}. \end{array}$$

To derive the response surface models, we utilized the replicate data provided in the simulation dataset. For the mean (${\hat{\mu }}_{i}$) and variance (${\hat{\sigma }}_{ii}^{2}$) functions, we adopted the models established by Kazemzadeh et al. [12]. However, for the covariance structure (${\hat{\sigma }}_{12}^{2}$), which is central to our study, we explicitly calculated the sample covariance for each design point using the four replicates and subsequently fitted a second-order regression model to these computed values.

To ensure the reliability of the optimization framework, the derived regression models must meet specific statistical criteria established in quality engineering literature. Following the guidelines suggested by Joglekar and May [13] and Montgomery [14], we adopted a significance level of p<0.05 and a minimum coefficient of determination $R^{2}>0.80$ as the thresholds for model adequacy. An $R^{2}$ value above 0.80 generally indicates that the regression model explains a substantial proportion of the variability in the response, which is essential for accurate prediction in robust design. If the regression models fail to meet these thresholds, it implies insufficient statistical power or excessive experimental noise. In such cases, the number of experimental replicates should be increased to reduce the standard error.

In this study, we utilized the dataset from Pignatiello [1] (n=4). To validate whether this sample size was adequate under our proposed criteria, we evaluated the statistical significance of the derived models. As shown in Table 3, all regression models achieved high coefficients of determination ($R^{2}>0.91$) and were statistically significant (p<0.05). Specifically, the covariance model (${\hat{\sigma }}_{12}^{2}$), a critical component of our framework, demonstrated an $R^{2}$ of 0.9726 with a *p*-value of 0.001. These results confirm that the sample size of n=4 provided sufficient statistical power to capture the underlying covariance structure accurately.

### 5.2. Formulation of Objective Functions

In this dataset, the target vector is τ=(103,73). Consistent with the definition in Section 3.1 where cost coefficients are determined by the economic impact of deviations, we adopt the specific values established in the study by Pignatiello [1]:

$$C=\left[\begin{array}{cc} 1.0 & 0.5\\ 0.5 & 2.5 \end{array}\right].$$

Based on these target values and cost values, the following functions are defined:

$${\hat{f}}_{1}\left(x\right)=\sum_{i=1}^{2}\sum_{j=1}^{2}c_{ij}\left({\hat{\mu }}_{i}\left(x\right)-\tau_{i}\right)\left({\hat{\mu }}_{j}\left(x\right)-\tau_{j}\right),\;{\hat{f}}_{2}\left(x\right)=\sum_{i=1}^{2}\sum_{j=1}^{2}c_{ij}\;{\hat{\sigma }}_{ij}^{2}\left(x\right).$$

By evaluating these functions across the experimental design space, we obtain the following statistical ranges:

$$U_{1}=192.28,\;L_{1}=3.643,\;\;\;\;\;U_{2}=63.87,\;L_{2}=0.306.$$

To generate a Pareto frontier, we employ an alternative GC-based method with the following objective function:

$$\min_{x}\;{\left(\frac{|{\hat{f}}_{1}\left(x\right)|}{192.28-3.643}\right)}^{p_{1}}+{\left(\frac{|{\hat{f}}_{2}\left(x\right)|}{63.87-0.306}\right)}^{p_{2}}.$$

### 5.3. Pareto Frontier Analysis

**Figure 5:**
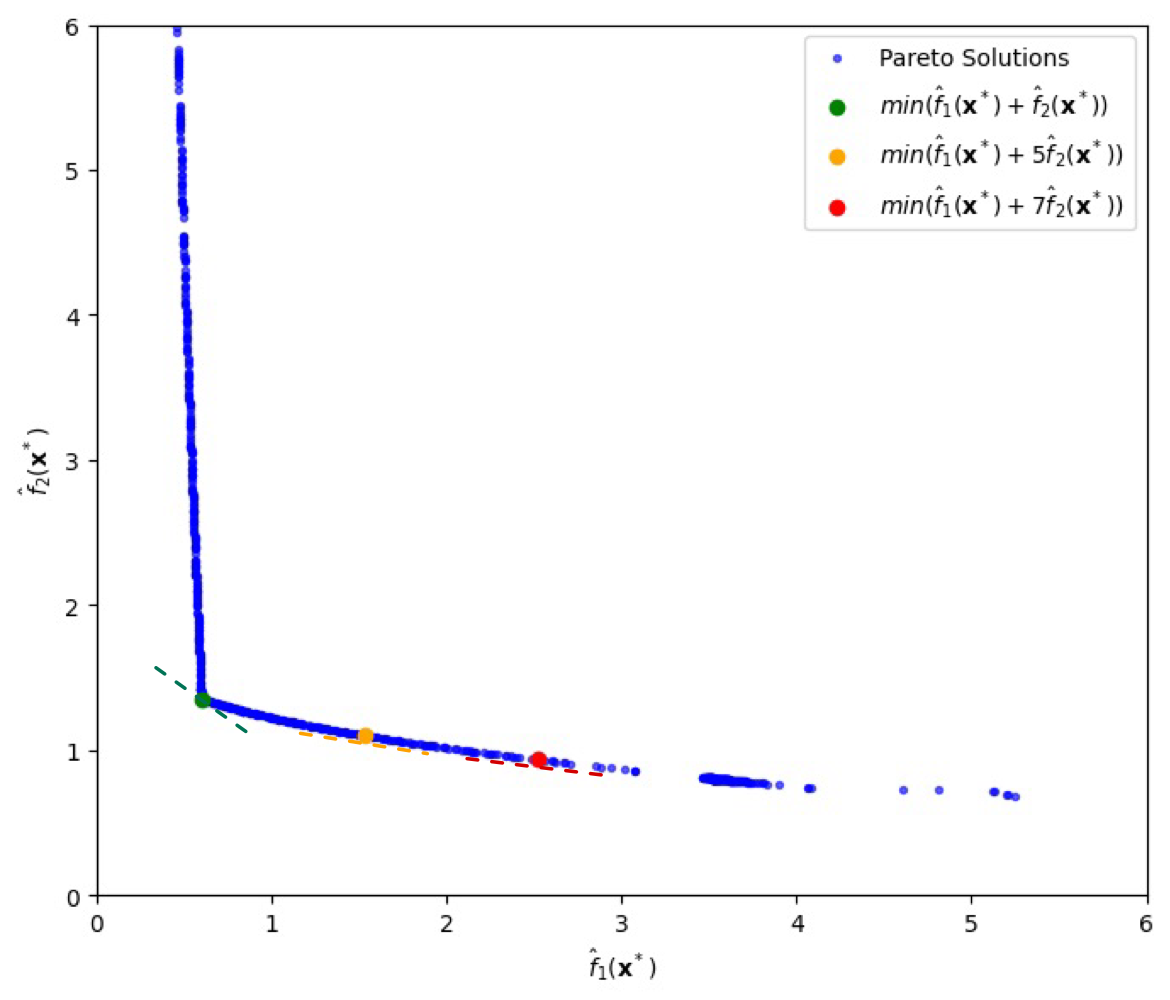
Pareto-front solutions for various variance-risk weights, λ=0,4,6, (depicted by the three circles coming from the tangent lines) in the simulation experiment.

Figure 5 shows a Pareto frontier generated by constraining the input variables to the range $-1\leq x_{i}\leq 1$, i=1,2,3, and varying the parameters $p_{1}$ and $p_{2}$ from 0.05 to 3 in increments of 0.05. Optimization is initialized from starting point (0,0,0).

When λ=0, we find the minimum total risk as follows:

$$T=1.95304,\;{\hat{f}}_{1}\left(x^{*}\right)=0.60067,\;{\hat{f}}_{2}\left(x^{*}\right)=1.35237,\;x^{*}=\left(1,0.71001,-1\right).$$

For λ=4, similarly using the front, we extract minimum total risk as follows:

$$T=7.02391,\;{\hat{f}}_{1}\left(x^{*}\right)=1.52971,\;{\hat{f}}_{2}\left(x^{*}\right)=1.09884,\;x^{*}=\left(0.52104,0.70273,-1\right).$$

Finally, when λ=6, minimum total risk becomes

$$T=9.05211,\;{\hat{f}}_{1}\left(x^{*}\right)=2.52681,\;{\hat{f}}_{2}\left(x^{*}\right)=0.93219,\;x^{*}=\left(0.17905,0.72327,-1\right).$$

The three dashed lines in green, yellow, and red shown in Figure 5 represent the tangents to the Pareto frontier at each solution that minimizes the weighted sum $T={\hat{f}}_{1}\left(x\right)+\left(1+\lambda \right){\hat{f}}_{2}\left(x\right),$ for λ=0,4,6, respectively.

### 5.4. Comparative Analysis with Sequential Strategies

A common practical approach to quality optimization is to adopt a sequential decision process. For instance, a field engineer may first minimize the bias term ${\hat{f}}_{1}\left(x\right)$, and then reduce the variance term ${\hat{f}}_{2}\left(x\right)$ among the solutions where bias is already minimized.

**Figure 6:**
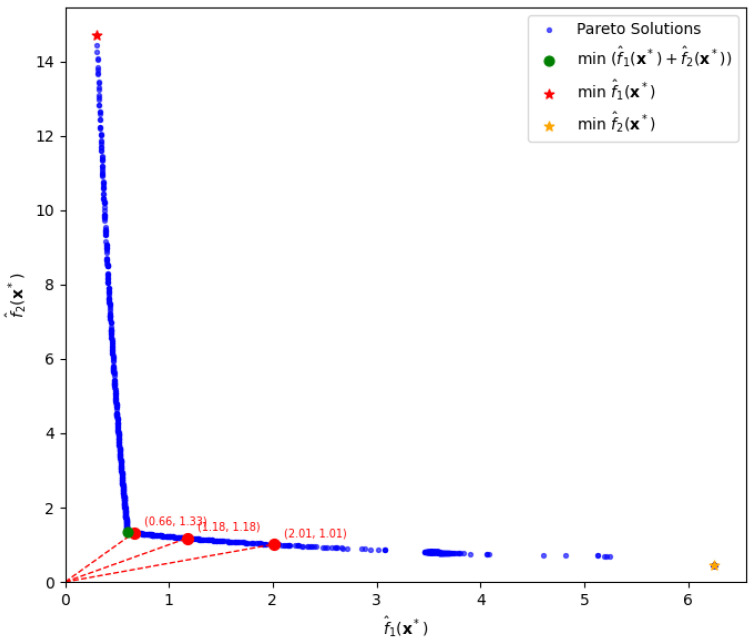
Comparison of decision strategies between sequential decisions depicted by two stars and Pareto-based decisions depicted by the green dot for the total risk, λ=0, and by the three red circles for various trade-off ratios of bias loss to variance loss, ${\hat{f}}_{1}\left(x^{*}\right):{\hat{f}}_{2}\left(x^{*}\right)$ being 1:2, 1:1, and 2:1.

To evaluate this practical strategy, we compare it against our proposed approach under the setting λ=0, where bias and variance are treated as equally important. The optimal solution minimizing the total risk, $T={\hat{f}}_{1}\left(x\right)+{\hat{f}}_{2}\left(x\right),$ is indicated by the green dot in Figure 6, yielding the minimum value T=1.95.

In contrast, when one adopts a sequential decision-making strategy, the outcomes are significantly suboptimal compared to the joint optimization approach. For instance, if one first minimizes the bias term ${\hat{f}}_{1}\left(x\right)$ and then evaluates the variance term at that point, the results are ${\hat{f}}_{1}\left(x^{*}\right)=0.30$ and ${\hat{f}}_{2}\left(x^{*}\right)=14.72$, yielding a total risk of $T={\hat{f}}_{1}\left(x^{*}\right)+{\hat{f}}_{2}\left(x^{*}\right)=15.02$ for λ=0. This point is highlighted by the red star, located at the upper-left side in Figure 6. On the other hand, if one first minimizes the variance term ${\hat{f}}_{2}\left(x\right)$ and then evaluates the bias at that point, the corresponding values are ${\hat{f}}_{1}\left(x^{*}\right)=6.25$ and ${\hat{f}}_{2}\left(x^{*}\right)=0.45$, resulting in a total risk of T=6.70. This point is highlighted by the yellow star, located at the lower-right side in Figure 6. Notice that the total risk, T=1.95, represented by the green dot in Figure 6, is far smaller than the values of T=15.02 and T=6.70 resulting from the two sequential decisions.

This comparison highlights that such a sequential optimization approach—whether prioritizing bias or variance—can lead to significantly worse solutions than those obtained by balancing both objectives jointly.

Furthermore, our approach enables flexible decision-making by allowing users to control the trade-offs between bias and variance via ratio settings. For example, we consider three target ratios: 1, 2, and 0.5. The corresponding solutions and function values are highlighted by the red circles in Figure 6 for direct comparison.

When the ratio ${\hat{f}}_{1}\left(x^{*}\right)/{\hat{f}}_{2}\left(x^{*}\right)$ is approximately 1, the optimal solution is $x^{*}=\left[0.673,\;0.702,\;-1\right]$, which yields ${\hat{f}}_{1}={\hat{f}}_{2}=1.18$. When the ratio is approximately 2, the solution becomes $x^{*}=\left[0.346,\;0.709,\;-1\right]$, resulting in ${\hat{f}}_{1}=2.01$ and ${\hat{f}}_{2}=1.01$. Lastly, for a ratio of 0.5, the solution is $x^{*}=\left[0.956,\;0.710,\;-1\right]$, with ${\hat{f}}_{1}=0.66$ and ${\hat{f}}_{2}=1.33$.

Consequently, our method simultaneously incorporates both bias and variance through a weighted formulation, enabling a more balanced and efficient optimization process. This approach identifies solutions with lower total risk, clearly demonstrating its effectiveness in achieving a superior trade-off between bias and variance. Additionally, our method provides the flexibility to choose mixing ratios between bias and variance terms, allowing for optimization that can be customized according to specific needs.

## 6. Conclusions

In this study, we proposed a novel approach for optimizing interrelated quality attributes by incorporating variance into the expected loss function. Leveraging an alternative GC-based method, we successfully constructed Pareto front to visualize the trade-offs between conflicting objectives. The effectiveness and practical utility of the proposed method were demonstrated through both simplified examples and a simulation study.

Nonetheless, constructing a comprehensive Pareto frontier requires the exploration of multiple starting points, and identifying all possible nondominated solutions remains a significant challenge. Future research should focus on developing systematic strategies for selecting initial points and benchmarking the proposed approach against existing multi-objective optimization techniques. Furthermore, as the complexity of the expected loss and variance functions increases, the associated computational burden also grows. Investigating simplified formulations or alternative structures to enhance computational efficiency would be a valuable direction for future work.

In addition, it is important to explore how this method can be extended to more realistic scenarios where only a subset of input variables—such as two or three out of many—can be actively controlled. Adapting our approach to such constrained optimization settings would further broaden its applicability.

Overall, this study contributes to the field of quality optimization by offering a new perspective on managing trade-offs in complex systems. The proposed method has the potential for application across various industries, and future extensions could include higher-dimensional problems and the integration of machine learning techniques for dynamic and adaptive optimization.

## Figures and Tables

**Table 1:** Simulation Data.

			$y_{1}$				$y_{2}$			
$x_{1}$	$x_{2}$	$x_{3}$	1	2	3	4	1	2	3	4
1	1	1	104.454	105.029	99.786	104.923	76.9003	77.0322	67.989	75.7691
1	1	−1	104.120	104.802	104.203	104.335	72.9878	74.2487	73.9371	73.2824
1	−1	1	98.732	99.357	102.842	94.235	67.0955	63.6112	68.647	62.4188
1	−1	−1	100.192	99.634	100.269	100.600	67.0264	66.1779	66.5758	67.9431
−1	1	1	103.145	106.959	107.620	103.440	71.6818	76.2657	77.4958	76.3739
−1	1	−1	106.078	105.642	105.670	105.393	72.9353	72.8508	72.5768	72.3754
−1	−1	1	113.515	111.121	112.854	106.666	68.2934	68.4693	68.9576	64.7051
−1	−1	−1	109.895	109.759	110.704	109.773	67.6974	67.2374	67.962	66.9268

**Table 2:** Statistical Summary of Simulation Data.

$x_{1}$	$x_{2}$	$x_{3}$	${\hat{\mu }}_{1}$	${\hat{\mu }}_{2}$	${\hat{\sigma }}_{1}^{2}$	${\hat{\sigma }}_{2}^{2}$	${\hat{\sigma }}_{12}^{2}$
1	1	1	103.54875	74.422675	6.5059	20.4381	6.4311
1	1	−1	104.365	73.614	0.0913	0.3887	0.1642
1	−1	1	98.2915	65.443125	14.1937	9.0943	5.6163
1	−1	−1	100.17375	66.4308	0.2062	0.5586	0.2039
−1	1	1	105.541	75.9543	4.1763	7.1282	4.5582
−1	1	−1	105.69575	72.684575	0.0975	0.0758	0.0707
−1	−1	1	111.539	67.60635	10.3569	3.3087	2.4789
−1	−1	−1	110.53275	67.4559	0.1971	0.2383	0.1715

**Table 3:** Statistical verification of the regression models (n=4).

Response	Regression Model	$R^{2}$	Model *p*-Value
Mean $y_{1}$	${\hat{\mu }}_{1}\left(x\right)$	0.9916	<0.001
Variance $y_{1}$	${\hat{\sigma }}_{11}^{2}\left(x\right)$	0.9699	<0.001
Mean $y_{2}$	${\hat{\mu }}_{2}\left(x\right)$	0.9866	0.004
Variance $y_{2}$	${\hat{\sigma }}_{22}^{2}\left(x\right)$	0.9180	0.012
Covariance	${\hat{\sigma }}_{12}^{2}\left(x\right)$	0.9726	0.001

## Data Availability

The data presented in this study are available on GitHub at https://github.com/KIM-sang-won/PFOVELIQ accessed on 4 February 2026.
